# Impact of organophosphate pesticides exposure on human semen parameters and testosterone: a systematic review and meta-analysis

**DOI:** 10.3389/fendo.2023.1227836

**Published:** 2023-10-24

**Authors:** Moses A. Hamed, Tunmise M. Akhigbe, Adetomiwa E. Adeogun, Oluwatosin B. Adesoye, Roland E. Akhigbe

**Affiliations:** ^1^ Department of Medical Laboratory Science, Afe Babalola University, Ado-Ekiti, Ekiti State, Nigeria; ^2^ The Brainwill Laboratory, Osogbo, Osun State, Nigeria; ^3^ Reproductive Biology and Toxicology Research Laboratory, Oasis of Grace Hospital, Osogbo, Osun State, Nigeria; ^4^ Department of Agronomy, Osun State Univeristy, Osogbo, Nigeria; ^5^ Department of Physiology, Ladoke Akintola University of Technology, Ogbomoso, Oyo State, Nigeria; ^6^ SickleLive Foundationo, Osogb, Nigeria; ^7^ SickleLive Foundation Research Laboratory, Osogbo, Nigeria; ^8^ State Specialist Hospital, Osogbo, Osun State, Nigeria

**Keywords:** endocrine disruptors, environmental toxicants, hormone imbalance, male infertility, organophosphate, pesticides, sperm, testosterone

## Abstract

**Background:**

Organophosphate (OP) pesticides have been associated with a decline in semen quality, although there are still considerable arguments about the magnitude of the association.

**Objective:**

This study provides a systematic review and meta-analysis of the impacts of OP pesticides on semen quality and male reproductive hormones.

**Methods:**

This study was conducted according to the Preferred Reporting Items for Systematic Reviews and Meta-Analyses (PRISMA) protocols. Strategic search was conducted using combined text words as search terms. The eligibility criteria were developed based on Population, Exposure, Comparator, Outcome, and Study designs (PECOS) framework. Relevant data were extracted, risk of bias was evaluated by The Office of Health Assessment and Translation (OHAT) tool, and certainty of evidence was assessed by the Grading of Recommendations Assessment, Development and Evaluation (GRADE) Working Group guidelines. Quantitative meta-analysis was performed by using Review Manager.

**Results:**

A total of 766 male subjects (349 exposed to OP pesticides and 417 unexposed controls) were included in the meta-analysis. There was no significant difference in the ejaculate volume, seminal fluid volume, sperm multiple anomaly index, sperm, and leukocytes levels of the OP-exposed subjects compared to the control. In addition, OP pesticides exposure did not significantly affect serum concentrations of FSH, LH, and testosterone in subjects who were exposed to OP pesticides compared to their unexposed counterparts. However, we found a significant reduction in the sperm count, sperm concentration, progressive sperm motility, total sperm motility, and normal sperm morphology of OP pesticides-exposed subjects compared to the unexposed subjects. However, after subtype and sensitivity analyses, exposure to OP pesticides did not reduce sperm count. Also, after sensitivity analysis, OP pesticides exposure did not alter progressive sperm motility.

**Conclusion:**

This study demonstrates that OP pesticides exposure reduced sperm count, concentration, total and progressive motility, and normal sperm morphology, possibly via a testosterone-independent mechanism.

## Introduction

An estimate of about one in six (approximately, 15%) couples are affected by infertility globally, and about 50% of this is due to male factor only and in combination with female factor (1–3). This has been associated with the global decline in sperm quality (4, 5), which occurs in concert with hormonal disruption (4). Testicular pathologies (such as cryptorchidism, testicular torsion and testicular cancer) (6–8), lifestyle factors, such as diets, smoking, energy dyshomeostasis and metabolic disorders (9–11), viral infections (12, 13), pharmaceuticals (14), and environmental toxicants, such as plasticizers and pesticides (15, 16) have been implicated in the pathogenesis of hormonal disruption and decline in sperm quality.

Although several human and experimental studies have shown that pesticides negatively alter normal physiological processes (17–20), they also act as endocrine-disrupting chemicals, leading to alterations in the normal hormonal milieu and reduced sperm quality (21, 22). Organophosphates are widely used pesticides for domestic and agricultural purposes (19, 20); however, they have been linked with endocrine disruption and poor sperm quality. A substantial body of evidence has demonstrated that organophosphate (OP) pesticides exert adverse effects on male reproductive hormones and sperm quality. However, most of these studies are on animal models and data on humans are limited with insufficient evidence to support this claim.

A cross-sectional study among Venezuelan farmer workers and unexposed control revealed that exposure to OP pesticides was negatively correlated with sperm concentration, morphology, and viability, while circulating testosterone, luteinizing hormone (LH) and follicle stimulating hormone (FSH) were not altered (23). In another cross-sectional study among Peruvian pesticide sprayers, observed a significantly lower ejaculate volume, sperm motility, and normal morphology as well as serum LH and testosterone levels among OP-exposed workers when compared with the control. Padungtod et al. (24) documented that exposure to OP pesticides among Chinese pesticide factory workers led to reduced sperm concentration and motility. Recio-vega et al. (25) however observed that OP pesticides exposure significantly reduced ejaculate volume and sperm count, but not motility and viability, while Hossain et al. (26) showed that OP pesticides exposure significantly reduced sperm concentration, motility, viability, and normal morphology. Unexpectedly, GhafouriKhosrowshahi et al. (27) reported that OP pesticides markedly reduced sperm count and motility but increased serum testosterone while ejaculate volume, semen pH and normal sperm morphology were not significantly affected. This is similar to the findings of Kamijima et al. (28) that observed a marked increase in serum testosterone levels among OP pesticides users. Fascinatingly, Multigner et al. (29) did not observe any significant difference in sperm parameters and serum male reproductive hormones in banana plantation workers that were exposed to OP pesticides and the unexposed counterparts, although they found significantly reduced testosterone levels in rats captured in the banana plantations compared with the control rats.

The World Health Organization (WHO) and the International Labour Organization (ILO) recommend a systematic review and meta-analysis of studies with estimates of the effects of occupational exposure with disease risk to estimate the burden of a particular exposure. In a meta-analysis, Giulioni et al. (30) demonstrated a significant reduction in ejaculate volume [Weighted mean difference (WMD) −0.47ml, 95%CI −0.69 to −0.25; *p* < 0.0001), sperm count (WMD-40.03, 95%CI −66.81 to −13,25; *p* = 0.003), concentration (WMD-13.69 x10^6^/mL, 95%CI −23, 27 to-4.12; *p* = 0.005) and motility (WMD −5.70%, 95%CI −12.89 to 1.50; *p* = 0.12) in OP pesticides-exposed workers. Although the negative association of organophosphates with spermatogenesis is noteworthy, the findings of Giulioni et al. (30) are with some shortcomings. First, some major studies were missing; only six studies were included in their study. This might have influenced their findings. In addition, Giulioni et al. (30) did not conduct a subtype and sensitivity studies to determine the source of heterogeneity. Moreso, the report of Giulioni and his colleagues did not appraise individual study included, thus the quality of evidence, publication bias, risk of bias, and certainty of evidence are unknown.

In a nutshell, human data on semen quality and male reproductive hormones in association with OP pesticides exposure are limited and inconsistent. Hence, the aim of this study was to analyze the association between OP pesticides exposure, sperm quality and testosterone levels through a systematic review and meta-analysis. Also, a comprehensive review of the associated mechanisms of OP pesticides-induced male reproductive dysfunction was presented. This study provides an in-depth understanding of the effect and associated mechanisms of OP pesticides on male reproductive function. The research question was structured according to PECOS statement (Population, Exposure, Comparators, Outcomes, and Study design); “what is the effect of OP pesticides exposure on human semen parameters and testosterone?”.

## Methods

### Literature search

This systematic review and meta-analysis was conducted on previously published articles that reported the impact of OP pesticides on semen quality and serum testosterone levels according to the Preferred Reporting Items for Systematic Reviews and Meta-Analyses (PRISMA) protocols (31). We conducted a systematic electronic search on CNKI, Cochrane Library, EMBASE, Pubmed/Pubmed Central, Scopus, Science Direct/Elsevier, and Web of Science database to identify published studies from inception to October 2022. The language and study type were not restricted. The search terms combined text words. The search terms for OP pesticides were: ‘OP pesticides’, ‘organophosphate chemical’, ‘organophosphate’, ‘OP chemical’, an ‘OP’. The search terms for semen parameters were: ‘sperm’, ‘sperm cell’, ‘spermatozoa’, ‘semen analysis’, ‘seminal fluid analysis’, ‘sperm parameters’, ‘sperm variables’, ‘sperm count’, ‘sperm concentration’, ‘sperm motility’, ‘sperm viability’, ‘sperm vitality’, ‘sperm morphology’, ‘semen volume’, ‘ejaculate volume’, ‘seminal pH’, ‘seminal leukocyte’. The search terms for male reproductive hormones were: ‘testosterone’, ‘luteinizing hormone’, ‘LH’, ‘follicle stimulating hormone’, ‘FSH’, and ‘male reproductive hormone’. All relevant articles and abstracts were retrieved. In addition, references cited in relevant articles were manually retrieved. The search strategy was pilot-tested and tested against benchmark papers.

### Selection of studies and validity assessment

The eligibility criteria for studies included in the meta-analysis were developed based on PECOS framework denoting the Population, Exposure, Comparator, Outcome, and Study designs of interest as stated below.

Inclusion criteria:

- Population: The population studied exclusively included male adults in their reproductive age group.
- Exposure: Studies that investigated the effect of one or more OP pesticides exposure, originating from domestic use or occupational exposure, for at least six months.
- Comparator: The studies must compare the OP-exposed individuals with normal age-matched unexposed male subjects.
- Outcomes: The association between OP pesticides exposure and semen parameters as well as serum testosterone levels is quantitatively reported. The mean and standard deviation could also be calculated from the provided data.
- Study design: The study design is either case-control, cohort, cross-sectional or ecological. These studies must be designed to adequately answer the research question “what is the effect of OP pesticides exposure on human semen parameters and testosterone?”.

Exclusion criteria:

- Population: Studies on male animal models and *in vitro* studies were not considered eligible
- Exposure: Studies on prenatal OP pesticide exposure were excluded. Also, studies on adult male exposure to pesticides other than OP pesticides were not included in this study.
- Comparator: Studies without unexposed healthy control adult males were excluded.
- Outcome: Studies that did not report numerical exposure variable and has higher risk of exposure misclassification and residual confounding were not included in this study. In addition, studies reporting health outcome by self-diagnosis were excluded.
- Study design: Studies that were not original studies (such as case reports, review articles, commentaries, letters, and editorials) were not considered eligible for inclusion.
- Conference abstract, thesis, preprint, or not peer reviewed/grey literature, literature review and systematic review articles were excluded.
- Retracted papers
- Studies that were not published in a peer-reviewed scholarly journal.
- Studies not written and published in English.

Two reviewers (ATM and AAE) independently screened the titles and abstracts of all the citations from the literature search. Relevant studies that met with the eligibility criteria were retrieved. The full text was analyzed if an equivocal decision was made on the basis of the title and abstract, and the final decision of eligible studies was made by reviewing the article. Disagreements were resolved by consensus or a third reviewer (HMA or ARE).

### Data extraction

The following details were extracted from each eligible study:

- Authors’ names
- The year the study was published
- Study design
- Country
- Type of OP pesticides
- Number of examined exposed and unexposed (control) subjects
- Age of subjects
- Duration of exposure to OP pesticides
- Outcomes/variables measured

### Quality of evidence assessment

The quality of each study included in the meta-analysis was assessed using the ErasmusAGE quality score for systematic reviews. The five domains assess included study design, study size, method of measuring exposure, method of measuring outcome, and analysis with adjustment. These domains were scores as: study design (0 = cross-sectional study, 1 = longitudinal study, 2 = intervention study), study size (0 = *<*50, 1 = 50 to 150, 2 = *>*150 participants), method of measuring exposure (0 = not reported, 1 = moderate quality exposure, 2 = good quality exposure), method of measuring outcome (0 = no appropriate outcome reported, 1 = moderate outcome quality, 2 = adequate outcome quality), and analysis with adjustments (0 = no adjustments, 1 = controlled for key confounders, 2 = additional adjustments for confounders) (32).

### Risk of bias assessment

The risk of bias (RoB) assessment was done by three reviewers (ATM, AAE, and HMA) for each study. Conflicts were resolved by the fourth reviewer (ARE). The Office of Health Assessment and Translation (OHAT) tool was used to assess the RoB for each included study. The six domains assess included selection bias, confounding bias, attrition/exclusion bias, deletion bias, selective reporting bias, and other bias. Each domain will be adjudged definitely low risk of bias, probably low risk of bias, definitely high risk of bias, or probably high risk of bias per study (33). Also, we visually assessed the total publication bias using the funnel plot generated by Review Manager (RevMan) software.

### Certainty of evidence assessment

The confidence in the body of evidence was rated using OHAT approach for systematic review and evidence integration for literature-based health assessment (34). This is based on the Grading of Recommendations Assessment, Development and Evaluation (GRADE) Working Group guidelines (35). Four descriptors were used to indicate the level of confidence; high, moderate, low, and very low (36).

### Meta-analysis

Quantitative meta-analysis was performed by using Review Manager (RevMan) software (version 5.4.1; the Nordic Cochrane Centre, the Cochrane Collaboration, 2012, Copenhagen, Denmark). Available data were analyzed in a meta-analysis, comparisons were made between the populations that were exposed to OP pesticides and the control groups and referred to as “exposed” and “unexposed”.

The standardized mean difference (SMD) of each reported variable was pooled from the included studies, which was identified with 95% confidence intervals (95% CIs). The P-value and I-square statistic (I^2^) in the pooled analyses were used to determine the heterogeneity of the studies, representing the percentage of total variation across studies. The summary estimate was analyzed in a random-effects model if the P-value was less than 0.1 or the I^2^-value greater than 50%; otherwise, a fixed-effects model was used. Visual symmetry of funnel plots was used to determine publication bias. The asymmetry of the funnel plot suggests possible publication bias.

### Subgroup and sensitivity analysis

To investigate possible sources of heterogeneity, we conducted subgroup analyses, excluding studies with exposure to unspecified organophosphates (which included 23 and 27). Also, studies with exposure to non-OP pesticides in addition to OP pesticides were excluded (which included 25 and 26). In addition, the study with participants older than 50 years (23) was excluded.

Sensitivity analyses were performed excluding the study with the largest weight, studies with at least one domain with “definitely high risk of bias” or “probably high risk of bias”, studies with low or very low confidence of evidence, studies with quality of evidence ≤ 5.

### Systematic review on mechanisms from animal and human *in vitro* studies

A comprehensive review of animal and human *in vitro* studies related to the effects and the associated mechanisms of OP pesticides and sperm quality and testosterone levels was also conducted.

## Results

### Study characteristics

**Figure 1:**
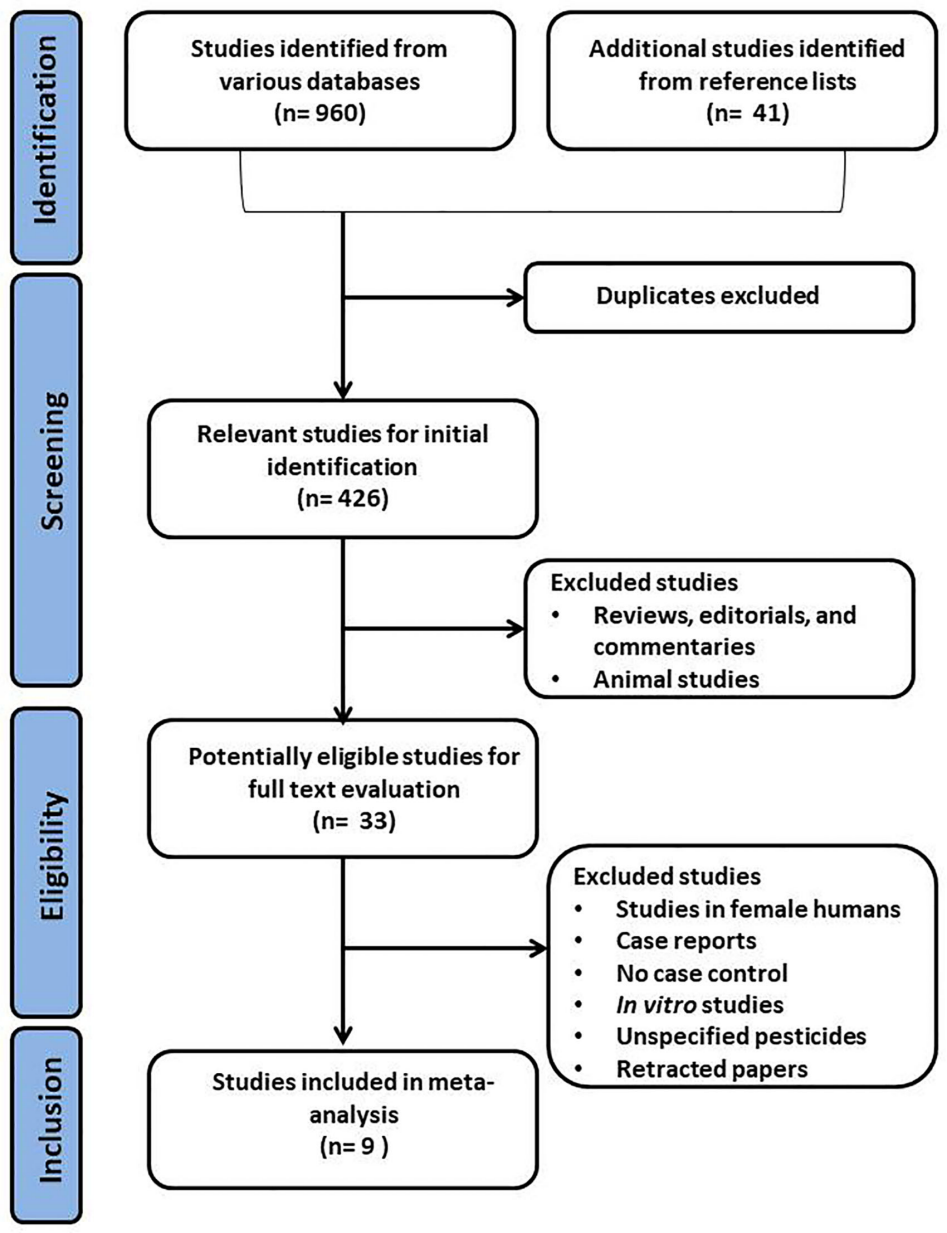
Preferred Reporting Items for Systematic Reviews and Meta-Analyses (PRISMA) flow chart of the selection process for eligible studies. .

Using the above-mentioned search strategy, 9 articles were identified as eligible for this study (**Figure 1**). Two of the studies were from China, and one each from Japan, Peru, France, Mexico, Malaysia, Venezuela, and Iran. Two of the studies did not specify the types of OP pesticides used, while the remaining 7 did. The characteristics of the selected studies are presented in **Table 1**. The study consisted of a total of 766 male subjects (349 exposed to OP pesticides and 417 unexposed controls).

**Table 1:** Eligible studies included in the meta-analysis that reported the effects of organophosphate pesticides on semen quality and male sex hormones.

References	Study design	Country	Type of OP	Examined population	Age (years)	Duration of exposure (years)	Outcomes/variables measured
37	Cross-sectional	China	Ethyl parathion, methamidophis	13 pesticide industry workers and 16 unexposed control	19-50 vs 22-47	3 to 24	Sperm concentration, total motility, and morphology
24	Cross-sectional	China	Ethyl parathion, methamidophis, methyl parathion	32 pesticide industry workers and 43 unexposed control	31±9 vs 30±8	12±9	Ejaculate volume, sperm count, concentration, total motility, progressive motility, and morphology
28	Cross-sectional	Japan	Fentothion, dichlorvos, chlorpyrifos, chlorpyrifos-methyl, diazinon, propetanphis,	15 pesticide industry workers and 16 unexposed control in summer; 14 pesticide industry workers and 15 unexposed control in winter	33.8±7 vs 34.5±7.5	0.5 to 25	Ejaculate volume, sperm concentration, count, viability, total motility, progressive motility, and morphology; FSH, LH, and testosterone
	Cross-sectional	Peru	Methamidophis	31 pesticide industry workers and 80 unexposed control	29.7±7.1 vs 32.8±7.6	–	Ejaculate volume, seminal fluid pH, concentration, count, viability, total motility, progressive motility, morphology, leukocyte, LH, FSH, testosterone
29	Cross-sectional	France	Cadusaphos, ethoprophos, isazophos, pyrimiphos-ethyl, terbulos	42 banana plantation workers and 45 unexposed control	34.8±6.3 vs 38.4±7.6	–	Ejaculate volume, seminal fluid pH, sperm concentration, count, total motility, progressive motility, morphology, multiple anomaly index, viability, FSH, LH, and testosterone
38	Cross-sectional	Mexico	Methylparathion, metamidiphis, endosulfan, dimethoate, diazinon	46 agriculture workers and 47 unexposed control	19-46 vs 18-47	–	Ejaculate volume, concentration, count, total motility, progressive motility, and viability
26	Cross-sectional	Malaysia	Malathion, paraquat	62 rural farmers and 90 unexposed control	–	–	Ejaculate volume, seminal fluid pH, sperm concentration, motility, morphology, leukocyte
23	Cross-sectional	Venezuela	Unspecified	64 agricultural workers and 35 unexposed control	18-52 vs 18-42	<2 to >5	Ejaculate volume, sperm, seminal fluid pH, concentration, count, motility, morphology, viability, multiple anomaly index
27	Cross-sectional	Iran	Unspecified	30 rural farmers and 30 unexposed control	20-40	–	Ejaculate volume, seminal fluid pH, sperm count, total motility, progressive motility, morphology, FSH, LH, and testosterone

OP, Organophosphate pesticides; * study that reported outcomes/variables in two seasons, summer and winter.

### Ejaculate volume

**Figure 2:**
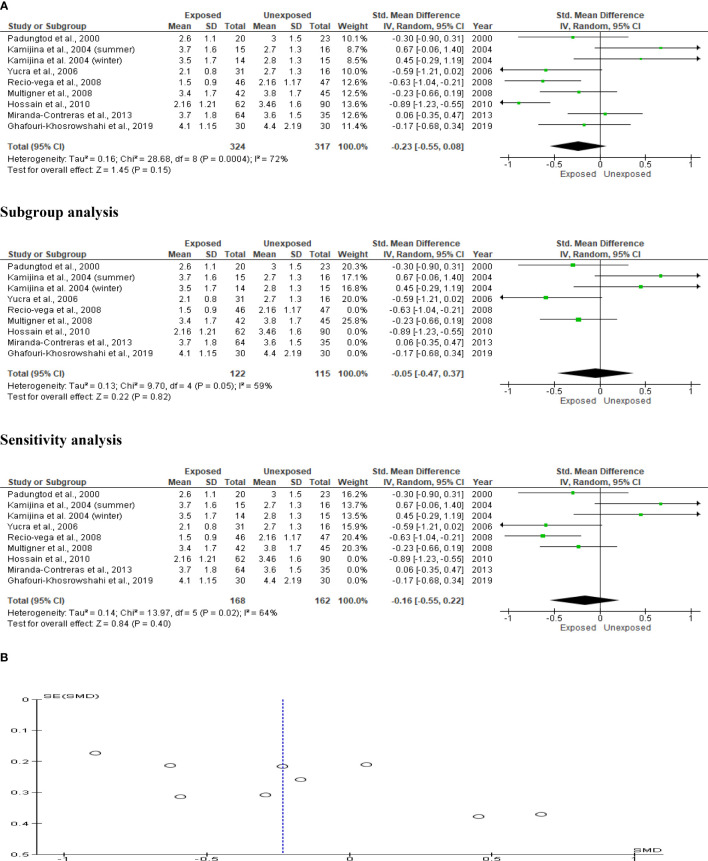
Forest plot **(A)** and publication bias funnel plot **(B)** of the effect of organophosphate pesticide exposure on ejaculate volume (mL).

Eight studies assessed the impact of OP pesticides exposure on ejaculate volume (324 in the exposed group and 317 in the unexposed control group). Kamijina et al. (28) examined this in two seasons; summer and winter. There was no significant difference in the ejaculate volume of the OP-exposed subjects compared to the control (SMD -0.23 [95% CI: -0.55, 0.08]; p=0.1), with the presence of significant inter-study heterogeneity (I^2 = ^72%; χ2 p=0.0004) (**Figure 2**). There was no significant publication bias. We also found out that OP exposure did not significantly alter ejaculate volume after subtype and sensitivity analyses were conducted (**Figure 2**).

### Seminal fluid pH

**Figure 3:**
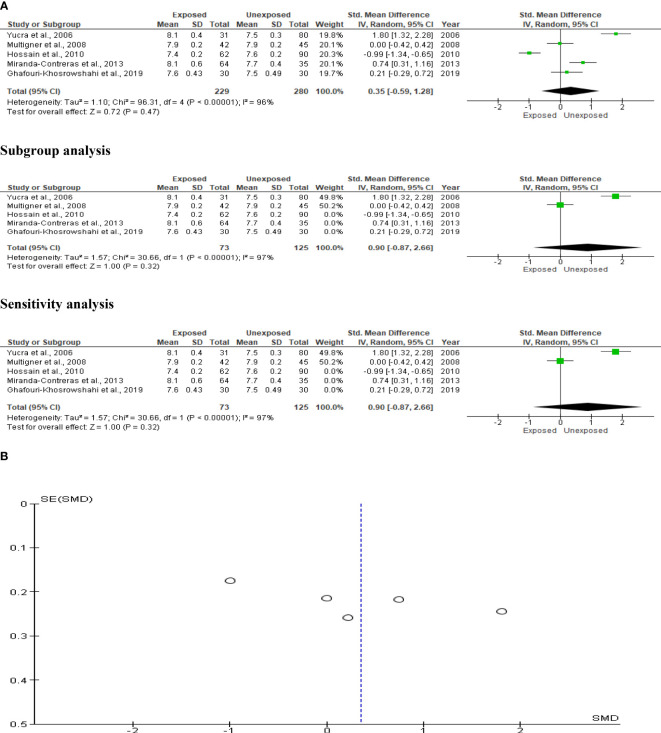
Forest plot **(A)** and publication bias funnel plot **(B)** of the effect of organophosphate pesticide exposure on seminal fluid pH.

Only five studies were included in the seminal fluid pH analysis, with a total of 229 exposed subjects and 280 unaffected controls. The analysis revealed that OP pesticide exposure had no effect on seminal fluid volume (SMD 0.35 [95% CI: -0.59, 1.28]; p=0.47), with significant inter-study heterogeneity (I^2 = ^96%; χ2 p0.00001). There was evidence of publication bias. After performing subtype and sensitivity analyses, we found that OP exposure had no impact on seminal fluid pH (**Figure 3**).

### Sperm count

**Figure 4:**
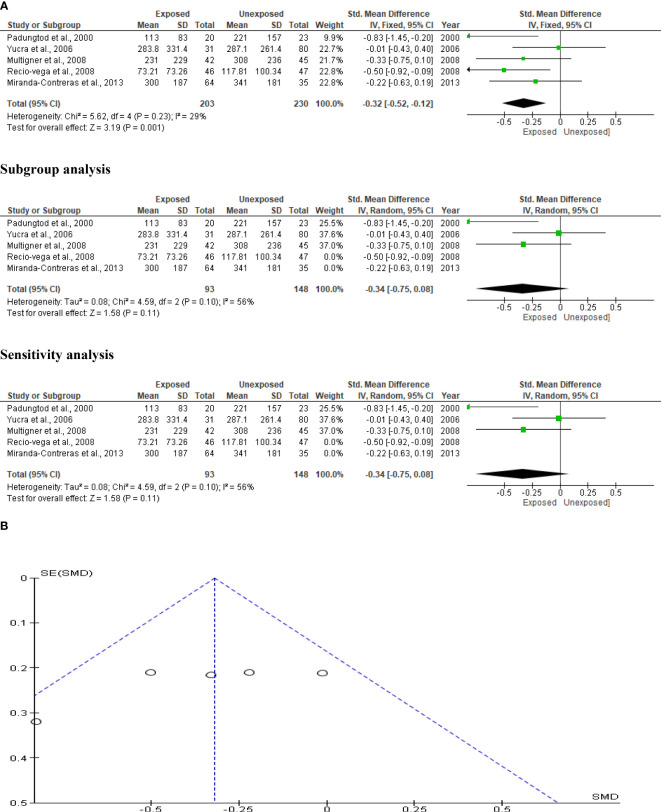
Forest plot **(A)** and publication bias funnel plot **(B)** of the effect of organophosphate pesticide exposure on sperm count (x 10^6^).

The analysis included five studies that reported data on OP pesticide exposure and sperm count. In a total population of 433 subjects, we found a significant reduction in sperm count of OP pesticide-exposed subjects compared to unexposed subjects (SMD-0.32 [95% CI: -0.52, -0.12] p=0.001), with no significant inter-study heterogeneity (I^2 = ^29%; χ2 p=0.23). There was no evidence of publication bias. However, exposure to OP pesticides did not substantially decrease sperm count after subtype and sensitivity analyses (**Figure 4**).

### Sperm concentration

**Figure 5:**
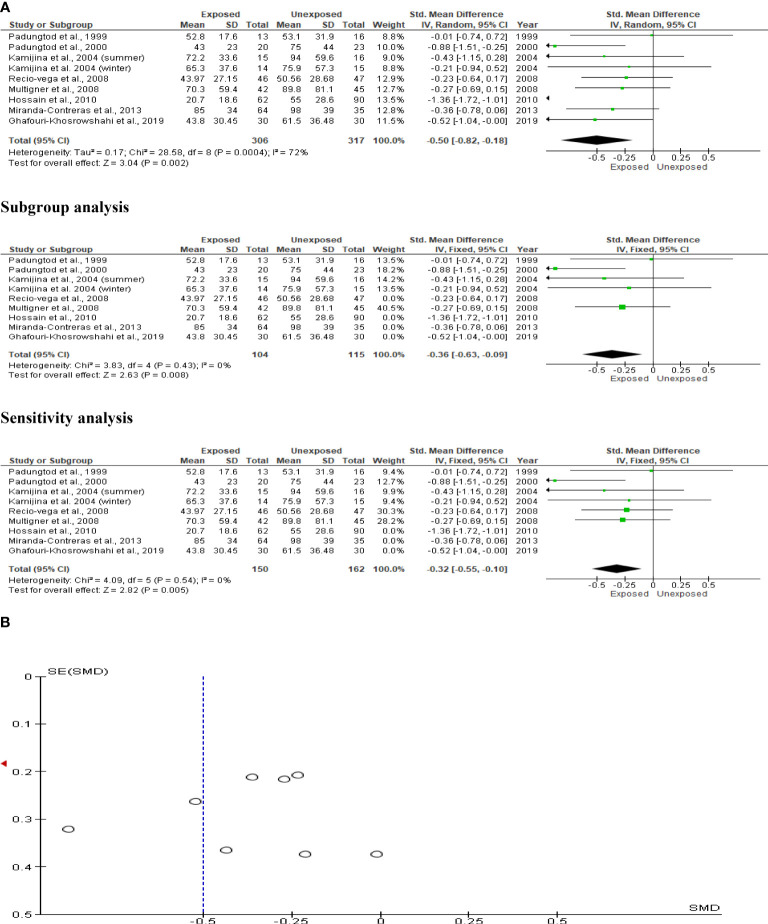
Forest plot **(A)** and publication bias funnel plot **(B)** of the effect of organophosphate pesticide exposure on sperm concentration (x 10^6^/mL).

Kamijina et al. (28) investigated this in two seasons, summer and winter, allowing them to analyze the results in 623 subjects (306 exposed subjects and 317 unaffected controls). The sperm concentrations of OP pesticide-exposed subjects were significantly lower than controls (SMD -0.50 [95% CI: -0.82, -0.18] p=0.002), with significant inter-study heterogeneity (I^2 = ^72%; χ2 p=0.0004). The publication bias was significant. Even after subtype and sensitivity analyses, the observed significant reduction in sperm concentration persisted (**Figure 5**).

### Progressive sperm motility

**Figure 6:**
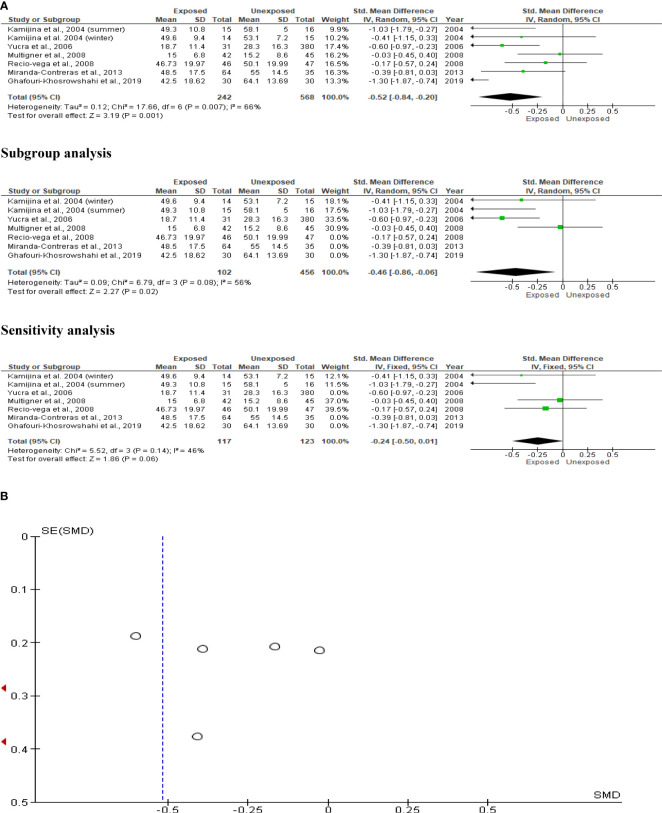
Forest plot **(A)** and publication bias funnel plot **(B)** of the effect of organophosphate pesticide exposure on progressive sperm motility (%).

The effect of OP pesticides on progressive sperm motility was studied in six studies, with Kamijina et al. (28) reporting findings in both the summer and winter seasons. There were a total of 810 subjects (242 exposed subjects and 568 unexposed controls). Subjects exposed to OP pesticides had significantly lower progressive sperm motility than those not exposed (SMD -0.52 [95% CI: -0.84, -0.20] p=0.001). Inter-study heterogeneity was significant (I^2 = ^66%; χ2 p=0.007). The Funnel plot was significantly asymmetrical, indicating that publication bias was present. The observed significant reduction in progressive sperm motility remained after sensitivity analysis, but it became comparable between the OP-exposed and unexposed groups (**Figure 6**).

### Total sperm motility

**Figure 7:**
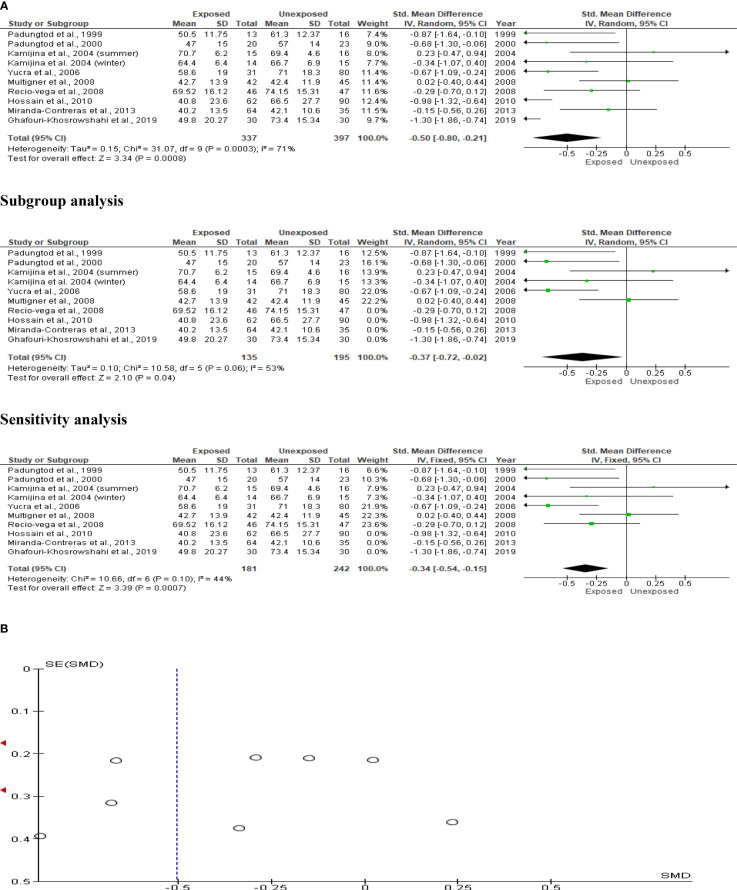
Forest plot **(A)** and publication bias funnel plot **(B)** of the effect of organophosphate pesticide exposure on total sperm motility (%).

The total sperm motility analysis comprised nine studies with a total of 734 participants (337 exposed and 397 controls). Kamijina et al. (28) investigated this in the summer in addition to the winter. Total sperm motility in OP pesticide-exposed individuals was significantly lower than in controls (SMD -0.50 [95% CI: -0.80, -0.21] p=0.0008). Inter-study heterogeneity was significant (I^2 = ^71%; χ2 p=0.0003). The asymmetry of the Funnel plot indicated significant publication bias. The observed significant reduction in total sperm motility persisted even after subtype and sensitivity analyses (**Figure 7**).

### Sperm morphology

**Figure 8:**
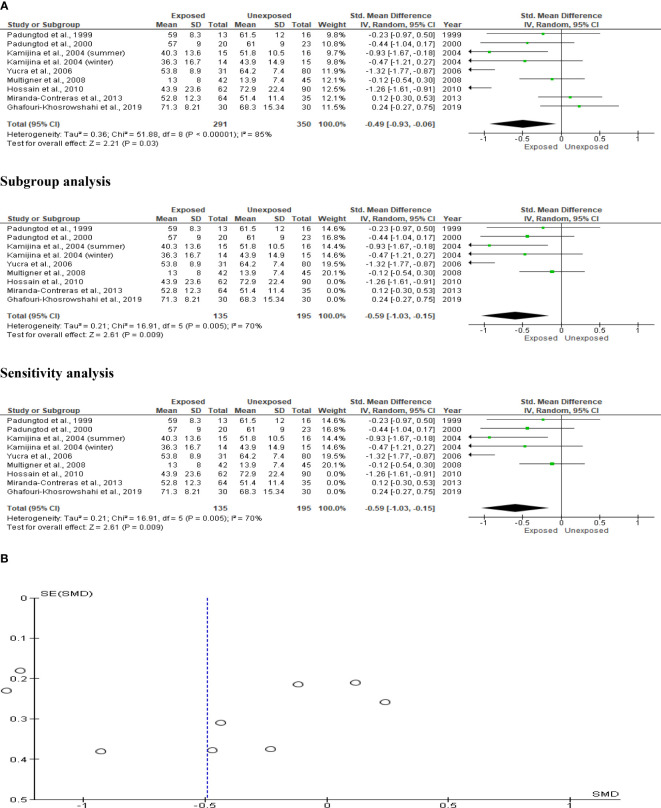
Forest plot **(A)** and publication bias funnel plot **(B)** of the effect of organophosphate pesticide exposure on normal sperm morphology (%).

The sperm morphology analysis comprised eight studies with a total of 641 men (291 exposed and 350 unexposed controls). Kamijina et al. (28) examined winter and summer variations of this. The exposed subjects had significantly fewer sperm with normal morphology (SMD -0.49 [95% CI: -0.93, 0.06] p=0.03) than the unexposed subjects. Highly significant inter-study heterogeneity was noted (I^2 = ^85%; χ2 p= 0.00001). Asymmetry in the funnel plot, which was discovered, is yet another indication of publication bias. This sperm morphology result was not influenced by subtype and sensitivity analyses (**Figure 8**).

### Sperm multiple anomaly index

**Figure 9:**
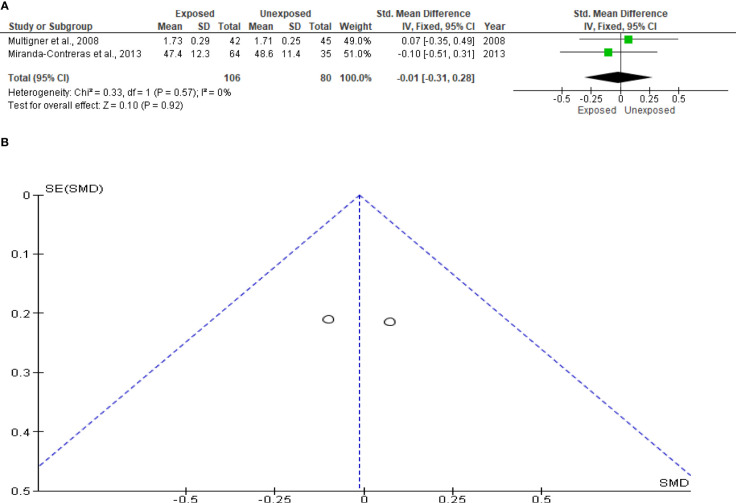
Forest plot **(A)** and publication bias funnel plot **(B)** of the effect of organophosphate pesticide exposure on sperm multiple anomaly index.

The analysis of the sperm multiple anomaly index only included two studies, totaling 106 subjects exposed to OP pesticides and 80 unexposed controls. When compared to the unexposed control, there was no discernible difference between exposure to OP pesticides and the sperm multiple anomaly index (SMD -0.01 [95% CI: -0.31, 0.28]; p=0.92). Additionally, there was no discernible inter-study heterogeneity (I^2 = ^0%; χ2 p =0.57). Confirming the absence of publication bias, funnel plot symmetry was also discovered (**Figure 9**).

### Sperm viability

**Figure 10:**
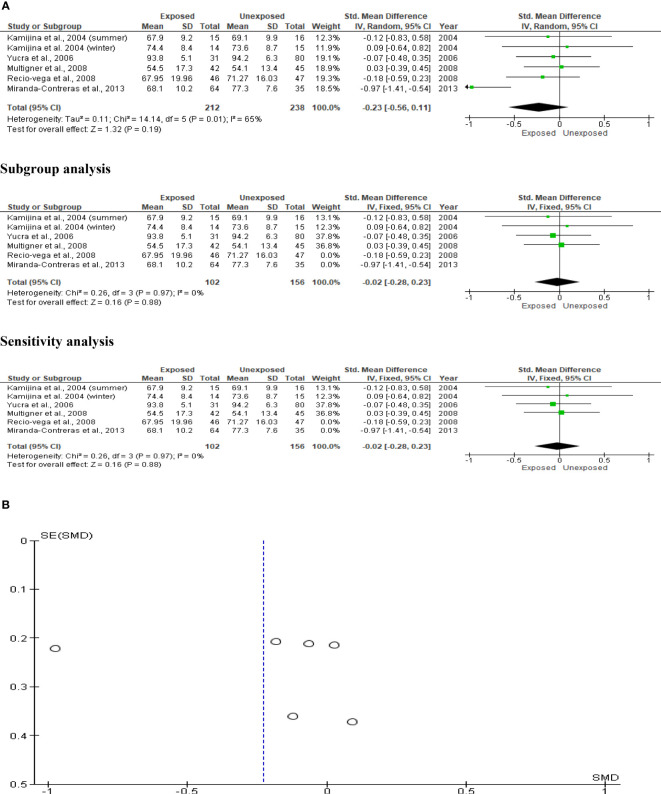
Forest plot **(A)** and publication bias funnel plot **(B)** of the effect of organophosphate pesticide exposure on sperm viability (%).

Sperm viability was examined in five studies involving a total of 212 exposed participants and 238 controls. Kamijina et al. (28) conducted an evaluation of this during the summer and the winter. When compared to unexposed controls, exposure to OP pesticides did not significantly affect sperm viability (SMD -0.23 [95% CI: -0.56, 0.11]; p=0.19). Inter-study heterogeneity was significantly evident in the analysis (I^2 = ^65%; χ2 p =0.01). Furthermore, funnel plot asymmetry was found, which is consistent with the presence of publication bias. This observation in sperm viability did not change after subtype and sensitivity analyses (**Figure 10**).

### Leukocyte level

**Figure 11:**
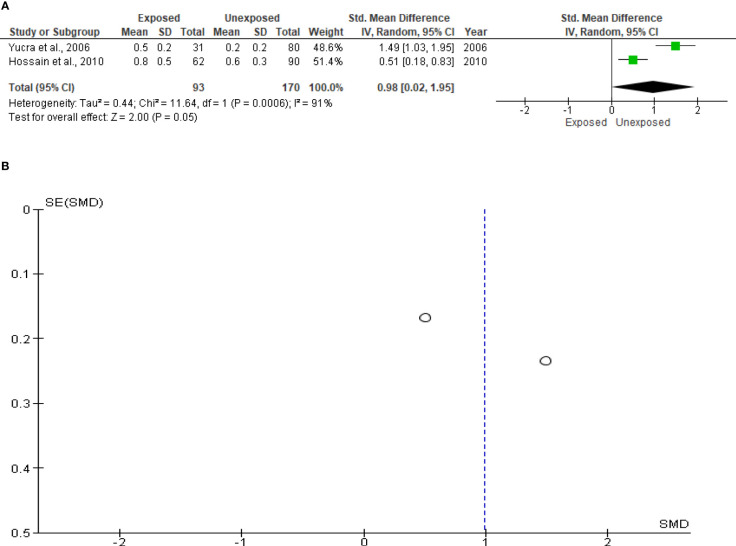
Forest plot **(A)** and publication bias funnel plot **(B)** of the effect of organophosphate pesticide exposure on leukocytes (x 10^6^/mL).

Only two studies with a total of 263 subjects (93 exposed and 170 unexposed controls) were included in the analysis of sperm leukocytes. Increased, but marginal, leukocyte levels were observed in OP pesticides-exposed subjects compared to the unexposed controls (SMD 0.98 [95% CI: 0.02, 1.95] p=0.05). Significant inter-study heterogeneity was observed (I^2 = ^91%; χ2 p=0.0006). The observed symmetry of the Funnel plot denoted no publication bias (**Figure 11**).

### Serum FSH

**Figure 12:**
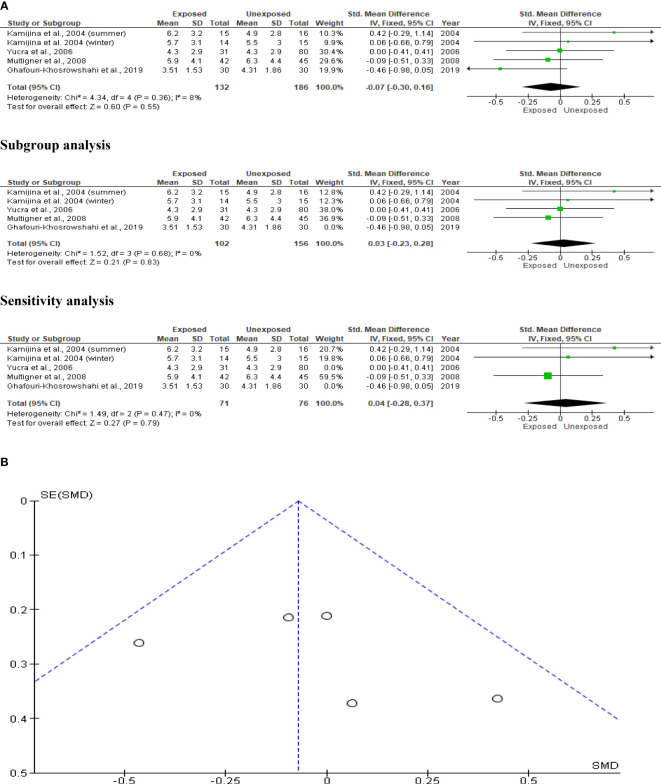
Forest plot **(A)** and publication bias funnel plot **(B)** of the effect of organophosphate pesticide exposure on serum FSH (IU/L).

The effect of OP pesticide exposure on serum FSH was studied in four studies, with Kamijina et al. (28) reporting results in both the summer and winter seasons. There were 318 subjects in total (132 exposed subjects and 186 unexposed controls). OP pesticides exposure did not significantly affect serum FSH concentrations in subjects who were exposed to OP pesticides compared to their unexposed counterparts (SMD -0.07 [95% CI: -0.30, 0.16] p=0.55). There was no significant inter-study heterogeneity observed (I^2 = ^8%; χ2 p=0.38). The Funnel plot was asymmetrical denoting the presence of publication bias. This observation in serum FSH did not change after subtype and sensitivity analyses (**Figure 12**).

### Serum LH

**Figure 13:**
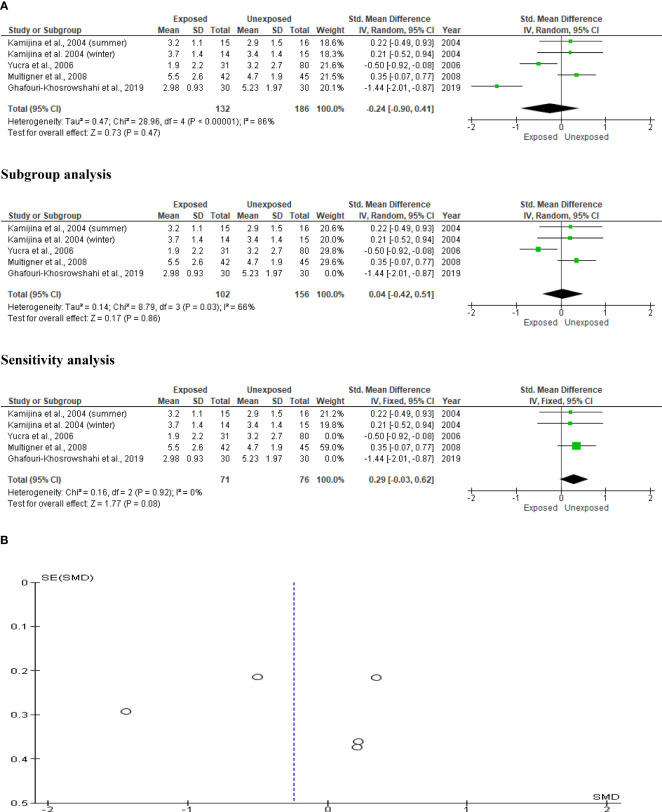
Forest plot **(A)** and publication bias funnel plot **(B)** of the effect of organophosphate pesticide exposure on serum LH (IU/L).

Kamijina et al. (28) evaluated this in two seasons, summer and winter, allowing analysis of this outcome in a total of 318 subjects (132 exposed subjects and 186 unexposed controls). OP pesticides exposure did not significantly alter circulating LH levels in OP pesticides-exposed subjects compared to the unexposed (SMD -0.24 [95% CI: -0.90, 0.41] p=0.47), with the presence of significant inter-study heterogeneity (I^2 = ^86%; χ2 p< 0.00001). The Funnel plot was asymmetrical, depicting publication bias. This observation in serum LH did not change after subtype and sensitivity analyses (**Figure 13**).

### Serum testosterone

**Figure 14:**
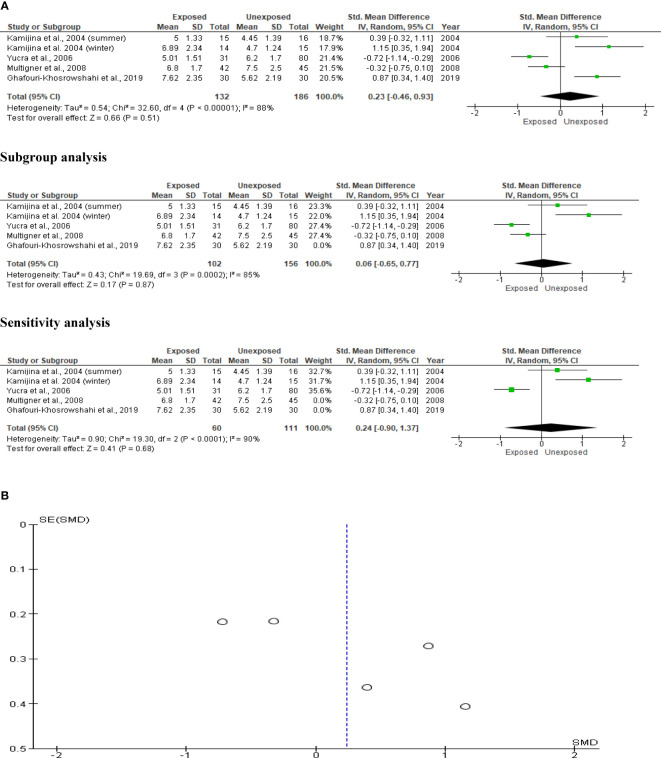
Forest plot **(A)** and publication bias funnel plot **(B)** of the effect of organophosphate pesticide exposure on serum testosterone (ng/mL).

The impact of OP pesticides on serum testosterone was examined in four studies, with Kamijina et al. (28) reporting findings from both the summer and winter seasons. In total, 318 subjects (132 exposed and 186 unexposed controls) were used. The analysis revealed that there was no significant difference in the circulating testosterone levels between the OP pesticides-exposed subjects and unexposed controls (SMD 0.23 [95% CI: -0.46, 0.93] p=0.51). Significant inter-study heterogeneity was observed (I^2 = ^88%; χ2 p<0.00001). The Funnel plot was asymmetrical denoting the presence of publication bias. This observation in serum testosterone did not change after subtype and sensitivity analyses (**Figure 14**).

## Discussion

### Key findings

This study reports a significant decline in sperm count, concentration, progressive and total motility, and normal morphology in individuals who were exposed to OP pesticides compared with unexposed controls. Although seminal fluid leukocyte levels were higher in OP pesticides-exposed individuals compared with unexposed controls using both studies that were included in this study (26), this was not significant when the studies were pooled together. In addition, it was observed that the circulating levels of LH, FSH, and testosterone were comparable between the OP pesticides-exposed and unexposed groups; this suggests that OP pesticides-induced low semen quality is testosterone-independent. Therefore, the data presented in this study provide a robust indication and strengthens available evidence that OP pesticides exposure lowers semen quality by reducing sperm count, concentration, motility, and normal morphology.

### Comparison to previous studies

The decline in sperm count observed in OP pesticides-exposed men is consistent with the findings of Padungtod et al. (24) and Recio-vega et al. (25), while our finding that OP pesticides-exposure cause reduced sperm concentration is also in agreement with the findings of Padungtod et al. (24) and Ghaouri-khosrowshahi et al. (27). In addition, these findings agree with observational cross-sectional studies that reported a negative association between OP pesticides and sperm count (22, 39–41). Sperm count is a measure of spermatogenesis, while sperm concentration is the most important parameter of testicular toxicity (42). Thus, based on the results on sperm count and concentration presented here, our data support the claim that OP pesticides impair spermatogenesis and exert toxic effects on testicular cells, especially germ cells. This forms an extension of the reports of Perez-Herrera et al. (43) that cells at all stages of spermatogenesis are a target of OP pesticides, and this effect may be mediated by paraoxonase (PON1) polymorphism.

In addition, our findings that OP pesticides significantly reduce sperm motility and normal sperm morphology align with some previous reports (24, 28, 37, 26, 27). These findings also agree with observational cross-sectional studies that documented a negative association between OP pesticides and sperm motility (22, 39, 41, 44, 45) and normal morphology (22, 40, 44–47). Since sperm function requires sperm motility, especially progressive motility (42), and sperm morphology is an important predictor of exposure to toxic substances and male factor infertility (48, 49). Our findings that OP pesticides reduces sperm motility and normal morphology confirm the spermo-toxic effect of OP, suggest that OP impairs sperm function, and also implicate OP in the incident male factor infertility. Although most of the human studies did not assess the likely mechanisms of action of the effect of OP pesticides on semen quality, GhafouriKhosrowshahi et al. (27) demonstrated that the impact of OP pesticides on semen quality may be due to its ability to increase nitric oxide, reduce total antioxidant capacity, and induce lipid peroxidation in the serum and seminal fluid.

Previous studies using animal models revealed that dichlorvos and diazinon, commonly used OP pesticides, exert spermotoxicity such as broken spermatozoa and reduced sperm motility (50, 51) as well as testicular toxicity (52). Suzuki et al. (53) demonstrated that OP pesticides-induced testicular and sperm toxicity was mediated via fatty acid amide hydrolase (FAAH), which plays key roles in spermatogenesis and sperm motility acquirement. Inhibition or downregulation of FAAH stimulates the cannabinoid signal, resulting in apoptosis of testicular cells like the Sertoli and Leydig cells by depriving the developing germ cells nutrients and hormonal signals needed for optimal development (54, 55). Exposure to Fenitrithion, an OP pesticides, induces testicular and sperm toxicity by inhibiting FAAH, although testicular AEA levels, which are usually modulated by FAAH inhibition, were not altered (53).

Studies have reported the direct testicular toxic effects of parathion, an OP pesticides, (56, 57), with an associated increase in abnormal sperm morphology, reduced chromatin quality, and increased apoptosis of germ cells. Parathion and its metabolite, paraoxon, also inhibit spermatogonial proliferation (38).

The toxic effects of OP pesticides have been linked with excessive generation of free radical (58; (59–61), which may alter the normal physiological function of the blood-testis barrier (62) producing covalent bonds with the occludens zone 2 (ZO2) (63) with multiple effects. This leads to lipid peroxidation of the sperm cell membrane, which is rich in polyunsaturated fatty acids (64), which exposed the protein content to denaturation and increases the susceptibility of the DNA in the nucleus to oxidative injury (65).

In the nucleus, OP chemicals modify the levels of mRNA encoding *Nrf2* and *OGG1*, which are important in the antioxidant buffering system and DNA repair (66–68). This may contribute, at least in part, to the observed reduction in the total antioxidant capacity of the seminal fluid in OP pesticides-exposed individuals (27), resulting in germ cell damage and consequent low sperm count and concentration. This may also promote ultrastructural abnormalities such as vacuolization, nuclear pyknosis, lipid droplets (50, 51, 66, 69), and increased DNA fragmentation (70). These may also explain the observed OP-induced sperm dysmotility and reduced normal sperm morphology.

### Limitations and strengths

This study has some limitations. First, it is likely, that the non-inclusion of non-English publications in the present meta-analysis and the scarcity of well-designed studies to be included might have limited the pooled sample size. This may inadequately explore the impacts of OP pesticides on semen quality and testosterone levels. In addition, the included studies are from a few countries, which may not necessarily be a good global representative. Also, the included studies did not report the exposure level of the studied population, which may affect the study outcome. Furthermore, the heterogeneity in the included studies resulted in the presence of outliers in the present meta-analysis; however, we were able to adjust for this with the statistical approach used. Nonetheless, owing to the completeness of our search, the present study seems to be the first robust study including all available case-control human studies reporting data on OP pesticides and semen quality and/or testosterone levels, avoiding many limitations of previous related studies. The present study also provides an extensive review of possible mechanisms using existing published data.

### Wider implications of our findings

OPs are pesticides, but also used as flame retardants and plasticizers, hence exposure to OPs is a common and global phenomenon. This rigorous and comprehensive meta-analysis reveals that OP pesticides exposure causes a significant decline in sperm count, concentration, total and progressive motility, and normal sperm morphology, which is consistent with direct suppressive and toxic effects of OP pesticides on spermatogenesis and sperm cells respectively, potentially affecting male fertility. However, testosterone levels remain unaltered despite a previous report by that OP pesticides significantly reduced testosterone levels.

The observed decline in sperm quality has wider implications beyond male fertility. Studies have linked low semen quality with socio-economic challenges (3, 14) and overall morbidity and mortality. Thus the observed decline in semen quality may exert ripple effects across the male lifespan. Our findings should, therefore drive a search for possible measures to prevent and ameliorate the impacts of OP pesticides on male fertility.

## Conclusion and future perspective

**Figure 15:**
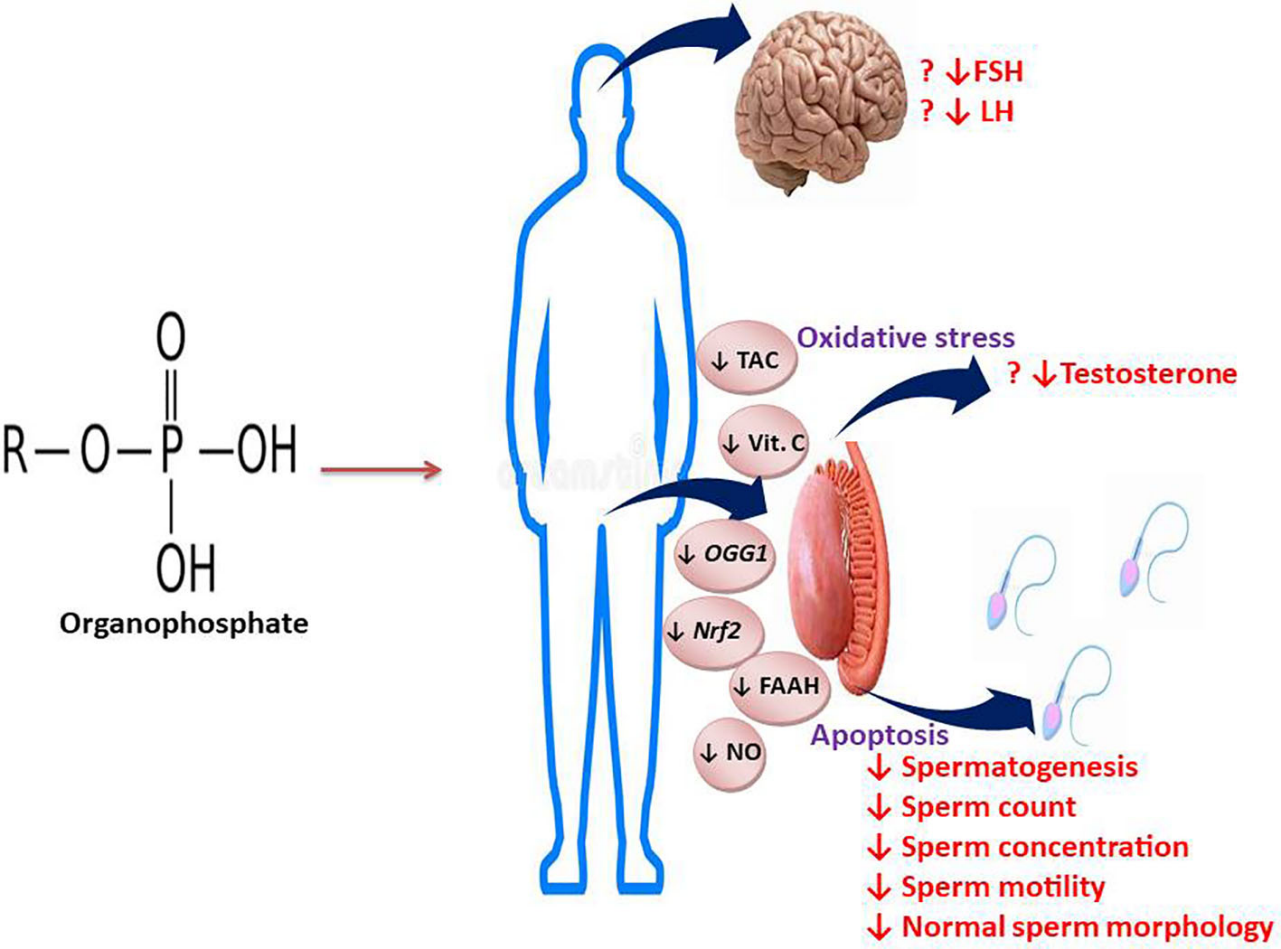
Graphical abstract illustrating the effect and mechanisms of action of OP on semen quality and male reproductive hormones.

The present comprehensive meta-analysis clearly demonstrates that exposure to OP pesticides causes reduced sperm count, concentration, total and progressive motility, and normal sperm morphology, possibly via a testosterone-independent mechanism (**Figure 15**). These findings strengthen existing evidence in the literature on the negative impacts of OP pesticides exposure on semen quality. Well-designed large case-control studies evaluating the effect and possible associated mechanisms of OP pesticides on semen quality are needed to reach more definitive conclusions. Also, possible measures that may prevent and/or ameliorate OP-induced low semen quality should be researched.

## Data availability statement

The original contributions presented in the study are included in the article/supplementary material. Further inquiries can be directed to the corresponding author.

## Author contributions

Conceptualization and design: MAH, TMA, and REA. Data curation: MAH, TMA, AEA, OBA, and ARE. Funding acquisition: MAH, TMA, AEA, OBA, and ARE. Investigation: MAH, TMA, AEA, OBA, and ARE. Methodology: MAH, TMA, AEA, OBA, and ARE. Project administration: MAH, TMA, AEA, OBA, and ARE. Supervision: MAH and REA. Validation: MAH, TMA, AEA, OBA, and ARE. Writing-original draft: MAH, TMA, and REA. Writing-review and editing and final approval: MAH, TMA, AEA, OBA, and ARE. All authors contributed to the article and approved the submitted version.
